# Energy Conservation for Internet of Things Tracking Applications Using Deep Reinforcement Learning

**DOI:** 10.3390/s21093261

**Published:** 2021-05-08

**Authors:** Salman Md Sultan, Muhammad Waleed, Jae-Young Pyun, Tai-Won Um

**Affiliations:** 1 Department of Information and Communication Engineering, Chosun University, Gwangju 61452, Korea; sultanmohammadsalman@chosun.kr (S.M.S.); mwk.uet@gmail.com (M.W.); 2 Department of Cyber Security, College of Science and Technology, Duksung Women’s University, Seoul 01369, Korea

**Keywords:** deep reinforcement learning, internet of things, target tracking, best sensor selection, energy consumption

## Abstract

The Internet of Things (IoT)-based target tracking system is required for applications such as smart farm, smart factory, and smart city where many sensor devices are jointly connected to collect the moving target positions. Each sensor device continuously runs on battery-operated power, consuming energy while perceiving target information in a particular environment. To reduce sensor device energy consumption in real-time IoT tracking applications, many traditional methods such as clustering, information-driven, and other approaches have previously been utilized to select the best sensor. However, applying machine learning methods, particularly deep reinforcement learning (Deep RL), to address the problem of sensor selection in tracking applications is quite demanding because of the limited sensor node battery lifetime. In this study, we proposed a long short-term memory deep Q-network (DQN)-based Deep RL target tracking model to overcome the problem of energy consumption in IoT target applications. The proposed method is utilized to select the energy-efficient best sensor while tracking the target. The best sensor is defined by the minimum distance function (i.e., derived as the state), which leads to lower energy consumption. The simulation results show favorable features in terms of the best sensor selection and energy consumption.

## 1. Introduction

In a 5G sensor network, a massive amount of data are handled via sensor devices in a large area. International Data Corporation (IDC) research states that 70% of companies will drive to use 1.2 billion devices for the connectivity management solution by 5G services worldwide [1]. The Internet of Things (IoT) is the future of massive connectivity under 5G sensor networks. Currently, the IoT is performing a vital role in collecting a large amount of data via numerous sensors in real-time applications [2]. Kevin Ashton initially coined the IoT concept in 1999 [1,3]. Sensor-based IoT devices can provide various types of services, such as health, traffic congestion control, robotics, and data analysis, which play a significant role in daily life assistance [4]. Target tracking is another critical area where the sensors can be utilized to collect the target real-time position and report it to a server with its relevant information. The practice of tracking one or multiple targets has vast applications in different research areas, such as object tracking (e.g., player, vehicle) [5,6,7], border monitoring to prevent illegal crossing, or battlefield surveillance [8], infrared target recognition [9,10].

In IoT target-tracking scenarios, tracking single or multiple targets can be realized using one or more sensors. However, it is impractical to utilize a single sensor for collecting the target position information owing to an extended area and will take increased computation with low tracking accuracy [11]. Therefore, it is pertinent to use multiple sensors, particularly in tracking applications. Energy consumption in sensor applications is a key task because of the sensor battery lifetime [11,12]. Moreover, it is unable to recharge the sensor battery in most cases. As a result, it is essential to efficiently reduce energy consumption because energy conservation leads to an increased battery lifespan. There are various energy consumption reduction methods used in recent years (e.g., clustering, support vector machine) [13,14]. However, large-scale functional implementation of these approaches precludes more time and resources.

**Figure 1:**
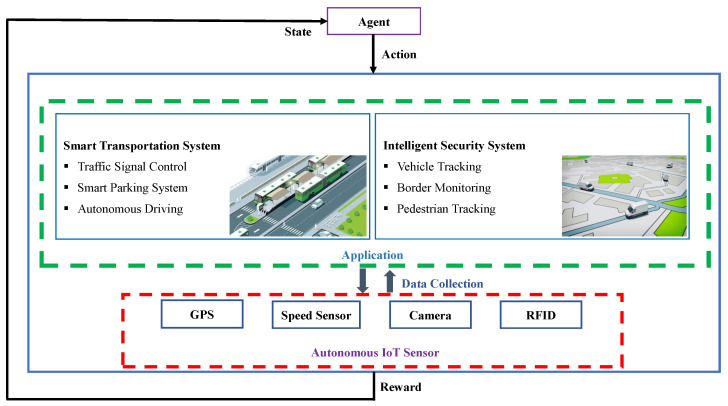
Autonomous IoT applications.

Reinforcement learning (RL) is a machine learning subfield that solves a problem without any predefined model. The RL agent learns the suboptimal policy by interacting with an unknown environment in real-time decision-based applications [15]. The use of RL comprises two main elements: action and reward. In any dynamic interactive environment, a precisely selected action will provide the best reward. Thus, providing the best outcome, based on current observations after acquiring a good reward in a real-time environment. However, a massive number of autonomous IoT sensors are employed to intelligently work with a dynamic environment to handle big data in next-generation 5G-based IoT applications (i.e., vehicle tracking, pedestrian tracking) [16]. Figure 1 shows some applications (e.g., smart transportation system, Intelligent Security System) including different types of sensors in the area of autonomous IoT. These autonomous IoT sensors interact and sense the environment to collect and send the relevant information to agent for taking the suboptimal action. The conventional RL algorithm (e.g., Tabular Q-learning) takes a higher time to handle this IoT environment because of large dimension sensor data [17].

Deep reinforcement learning (Deep RL) is an extended version of the conventional RL algorithm to overcome iteration complexity in any large dynamic and interactive environment [18]. Deep neural network (described as Q-approximator in this paper) is the main feature of Deep RL, predicting a suboptimal action from a specific state. In an autonomous IoT target tracking system, Deep RL can be deployed to the sensor devices to minimize the overall system computational complexity and energy consumption [17,19]. Moreover, there are different kinds of Q-approximators used in the Deep RL method to solve the energy consumption problem. Dense and long short-term memory (LSTM)-based Q-approximators are frequently utilized to increase energy efficiency in time-series environments [20,21]. Note that the LSTM Q-approximator is more suitable than the dense Q-approximator because of long-term dependencies in an IoT target tracking environment. The long-term memory features regulate the essential information sequentially (i.e., time-dependent) to achieve better performance in the learning period [22,23,24].

In this study, we proposed a novel Deep RL framework that predicts the suboptimal energy-efficient sensor to track the target in IoT tracking applications. Our proposed system utilizes an LSTM deep Q-network (LSTM-DQN) as Q-approximator. Moreover, a data pre-processing approach is used for better state representation before applying LSTM Q-approximator. The data pre-processing (e.g., normalization, feature selection) is significant for achieving stable LSTM Q-approximator [25,26]. In this paper, we use mini-max normalization into our designed state space to improve LSTM Q-approximator performance. Furthermore, we also study epsilon-greedy and softmax action-selection strategies [27] in our proposed target tracking environment. However, the epsilon-greedy method has faster improvement and convergence ability than the softmax method in our action space. Therefore, we proposed an LSTM-DQN-epsilon-greedy method and compare it with LSTM-DQN-softmax, Dense-DQN-epsilon-greedy, and Dense-DQN-softmax approaches in terms of average cumulative rewards, loss convergence, average sensor selection accuracy, and average cumulative energy consumption.

The remainder of this paper is organized as follows. A description of the related work is provided in Section 2. Section 3 presents the system preliminaries. Section 4 and Section 5 show our proposed LSTM-DQN-epsilon-greedy algorithm and numerical results, respectively, for a detailed comparison. Finally, Section 6 presents the conclusion and future directions of the research work.

## 2. Related Work

In recent years, researchers have been working and investing much of their time to solve the problem of excessive energy consumption in tracking-based applications. Below, applications based on the respective techniques from background studies are presented.

### 2.1. Tracking Application Based on Information-Driven Approaches

Information-driven is a collaborative sensing technique for various target tracking applications, where each deployed sensor is responsible for collaborating with other deployed sensors to collect moving target information [28]. Information-driven methods were first proposed in terms of collaborative sensor selection via the information utility function [29]. In this information-driven sensor selection method, the authors considered different Bayesian estimation problems (e.g., entropy and Mahalanobis distance-based utility measurements) to determine which sensor would track the moving target. Wei et al. [30] proposed a dual-sensor control technique based on the information utility function in a multi-target tracking application. In this work, the authors used the posterior distance between sensor and targets (PDST) function to minimize the distance between sensors and targets, which helped the sensors directly drive the targets. Ping et el. in [31] used a partially observed Markov decision process (POMDP) to select suboptimal sensors for tracking multiple targets. The POMDP sensor selection approach is implemented by maximizing the information gain via a probability hypothesis density (PHD)-based Bayesian framework. Although the techniques proposed in [29,30,31] illustrated good tracking results, there is a limitation in choosing an energy-efficient sensor to make their model work in an intelligent manner to reduce the computational complexity.

### 2.2. Machine Learning-Based Techniques for Tracking Application

Machine learning is an excellent technique to overcome the computational complexity issue in any complicated engineering problem because it is a self-learner, and it does not need to be reprogrammed [32,33,34,35]. Based on background studies, there are three types of machine learning approaches (i.e., supervised, unsupervised, and reinforcement learning), which have been intelligently utilized for energy optimization. The study of supervised learning techniques is beyond the scope of this research.

#### 2.2.1. Unsupervised Learning-based Clustering Approaches

To address the energy consumption problem, Hosseini and Mirvaziri in [36] introduced a dynamic K-means clustering-based approach to minimize the target tracking error and energy consumption in wireless sensor networks (WSNs). The proposed technique uses a tube-shaped layering method for the sensor nodes to reduce energy dissipation during target tracking. In addition, Tengyue et al. [37] employed a clustering algorithm to control the sensor energy, which detected the target in a real-time mobile sensor network. They used the k-means++ algorithm to separate the sensor nodes into sub-groups. The k-means++ separated the sensor nodes, which carried a higher weighted probability for target detection, and the remaining unnecessary sensors remained in sleep mode to save energy consumption. Juan and Hongwei in [38] proposed another clustering approach to balance energy in terms of multisensory distributed scheduling. Their work used the energy-balance technique to control the activation and deactivation modes of communication modules. They employed a multi-hop coordination strategy to decrease energy consumption. However, these types of unsupervised techniques are time-consuming to address because of the lack of available prior data labeling [34].

#### 2.2.2. Reinforcement Learning Approaches

Sensor scheduling is a promising approach for reducing energy consumption in many tracking applications. Muhidul et al. in [39] proposed a cooperative RL to schedule the task of each node based on the current tracking environment observation. The proposed method helped the deployed sensor nodes cooperate by sharing the adjacent node information during tracking. They applied a weighted reward function that combined both energy consumption and tracking quality matrices to improve the sensor node task scheduling at a particular time. Moreover, transmission scheduling is another necessary task in which Deep RL can be applied. Jiang et al. in [40] proposed an approximation technique for transmitting packets in a scheduling manner for cognitive IoT networks. Their DQN model utilized two parameters (i.e., the power for packet sending via multiple channels and packet dropping) to enhance the system capacity in throughput terms. They used a stacked auto-encoder as a Q-function approximator that mapped the policy to maximize system performance via a utility-based reward technique. However, they exploited the action using a comprehensive index evaluation method in a single relay to sync transmission.

To reduce IoT device energy consumption, Mehdi et al. [41] employed a Deep RL technique to learn an optimal policy for indoor localization problems in IoT-based smart city services. They deployed a semi-supervised technique to classify unlabeled data and integrated classified data with label data. They used iBeacons to provide a received signal strength indicator (RSSI) as an input for a semi-supervised Deep RL model, which consists of a variational autoencoder neural network Q-learning technique to enhance indoor localization performance. In [27], the authors used two Deep RL methods (e.g., DQN and DDPG) to adjust the activation area radius so the system can minimize the average energy consumption in terms of vehicle-to-infrastructure (V2I) technology-based tracking applications. They also used two action selection strategies (e.g., epsilon-greedy and softmax) to determine the activation area radius.

The Deep RL method has not been widely applied for energy saving in IoT target tracking applications, particularly in energy-efficient sensor selection approaches. Intelligently selecting the appropriate sensor to track the target is challenging because the target position varies over time, creating tracking environment uncertainty. In this case, the DQN-based Deep RL is a sophisticated method because it has the best learning capability when interacting with an uncertain dynamic environment. In DQN, selecting a Q-approximator for the tracking environment is vital for obtaining improved learning performance. Therefore, we utilized the LSTM Q-approximator to predict the suboptimal decisions (i.e., sensor selection) based on sequential information (i.e., target position) with the assistance of different gate operations.

Our study is based on a discrete action space, which means that the proposed LSTM Q-approximator selects the most energy-efficient sensor among a finite set of sensors. Authors in [27] showed epsilon-greedy and softmax-based action selection methods for the discrete action space. The epsilon-greedy-based sensor-selection technique presented improved efficiency compared to the softmax technique in the simulation results. Thus, we proposed the LSTM-DQN method with epsilon-greedy action selection (described as LSTM-DQN-epsilon-greedy in this study) in a target tracking environment to select the best sensor for maximum energy conservation. Table 1 represents a comparison of different existed RL methods to reduce the energy consumption of the sensor.

## 3. Preliminaries

### 3.1. System Overview

**Figure 2:**
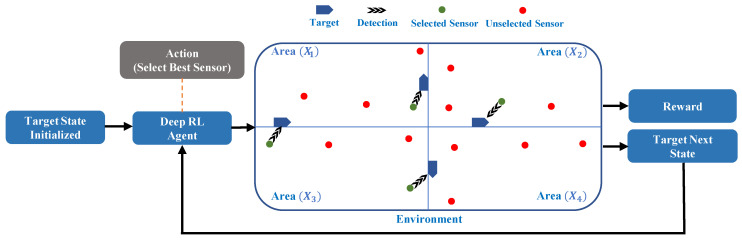
Deployed sensors for tracking target-based environment.

Figure 2 illustrates the tracking environment where multiple sensor devices represented as *S* = {$S_{1},S_{2},\ldots ..,S_{D}$} are deployed at different positions to observe the moving targets, *T* = {$T_{1},T_{2},\ldots ..,T_{L}$}, where *L* is the number of targets moving in the test area. The area consists of subareas *X* = {$X_{1},X_{2},\ldots ..,X_{N}$}, where *N* is the number of subareas.

In this study, our proposed LSTM-DQN-epsilon-greedy scheme allows one sensor to track a single target at time *t* in a particular area, which eventually leads to tracking *T* targets in *N* subareas. For instance, the selected sensors shown in green detect the targets, as shown in Figure 2. The remaining sensors remained unselected to minimize energy consumption.

For suboptimal sensor selection, our proposed LSTM-DQN-epsilon-greedy-based IoT tracking system tracks more than one target simultaneously in four subareas $X_{1}$, $X_{2}$, $X_{3}$, and $X_{4}$, as shown in Figure 2, thus allowing the system to track all *T* targets in the first attempt. If we apply a single DQN algorithm for all *N* subareas, there is a possibility of not achieving the required goal because when the system interacts with a large area, the sensor selection space is more complicated to utilize the algorithm for effective simultaneous tracking more than one target.

To select the best sensor, it is imperative to estimate the distance between the moving target and the sensors. A sensor with the minimum distance to the target location was selected. However, in any practical scenario, the sensor has some noisy (i.e., Gaussian noise) measurements; thus, it can not collect the target position precisely. This study considers that our target tracking environment is linear, including normally distributed or Gaussian process noise and some measurement errors. Kalman filter is suitable for any linear environment along with Gaussian noise to predict the target information with more precision [42,43,44]. Moreover, because of having linear features, the Kalman filter does not require significant memory except knowing only the prior state, which assists in predicting the target state over time [44]. Therefore, For the accurate measurement in a linear and noisy environment, the Kalman filter was used to localize the target position.

### 3.2. Kalman Filter

The Kalman filter estimates the current system state from a series of noisy measurements, which is useful in tracking applications [42,45,46,47]. The Kalman filter is a recursive estimator based on Bayesian filter theory that can compute the target state along with the uncertainty [43,44]. The system has two significant steps: prediction and updating. Various essential Kalman filter parameters are listed in Table 2.

The initial state matrix $\alpha_{0}$ indicates the early stage target observation and consists of four key information pieces such as the x- (*x*) and *y*-axis (*y*) positions, velocity along the x- (vx) and *y*-axis (vy). In general, the covariance process measures the variation in random variables. The covariance for the four random variables is defined as follows:

(1) $$\sigma \left(x,y,vx,vy\right)=\frac{1}{n-1}\;\sum_{i=1}^{n}\left(x_{i}-\bar{x}\right)\left(y_{i}-\bar{y}\right)\left(x_{i}^{\prime }-\bar{vx}\right)\left(y_{i}^{\prime }-\bar{vy}\right),$$

where *n* is the number of samples, and the covariance matrix is defined as ${\sigma \left(x,y,vx,vy\right)}^{T}$. The initial state $\alpha_{0}$ and process covariance matrices $P_{0}$ are expressed as,

(2) $$\alpha_{0}=\left[\begin{array}{c} x\\ y\\ vx\\ vy \end{array}\right]$$

(3) $$P_{0}=\left[\begin{array}{cccc} \sigma^{2}x & \sigma_{x}\sigma_{y} & \sigma_{x}\sigma_{vx} & \sigma_{x}\sigma_{vy}\\ \sigma_{y}\sigma_{x} & \sigma^{2}y & \sigma_{y}\sigma_{vx} & \sigma_{y}\sigma_{vy}\\ \sigma_{vx}\sigma_{x} & \sigma_{vx}\sigma_{y} & \sigma^{2}vx & \sigma_{vx}\sigma_{vy}\\ \sigma_{vy}\sigma_{x} & \sigma_{vy}\sigma_{y} & \sigma_{vy}\sigma_{vx} & \sigma^{2}vy \end{array}\right].$$

In the Kalman filter, the prediction step estimates the current predicted state $\alpha_{k}$ and the process error covariance matrix $P_{k}$, which are expressed as,

(4) $$\alpha_{k}=X\alpha_{k-1}+YAcc_{k}+N_{k\alpha },$$

(5) $$P_{k}=X\left(P_{k-1}X^{T}\right)+YAcc_{k}+N_{kp}\;,$$

where $\alpha_{k-1}$ and $P_{k-1}$ denote the previous state and process error covariance matrices, respectively. The variable *X* represents the state transition matrix for the previous state $\alpha_{k-1}$, and *Y* is the input transition matrix for the control vector. The $Acc_{k}$ in (6) shows the acceleration of the moving target, given as,

(6) $$YAcc_{k}={\left[\begin{array}{cccc} \frac{1}{2}\Delta T^{2}ax & \frac{1}{2}\Delta T^{2}ay & \Delta Tax & \Delta Tay \end{array}\right]}^{T},$$

where ΔT represents the time for one cycle, while ax and ay are the acceleration control variables. In the updated step, we estimate a new measurement $M_{k}$ for state prediction at time step *k*. The Kalman gain *G* is one of the main features in the Kalman filter method, which gives the ratio of the uncertainty of error in prediction and measurement state [42]. Moreover, Kalman gain indicates how much the prediction state of the target should be precise. If the value of Kalman gain is increased gradually, which means the uncertainty error of the measurement is small, and the value of the Kalman gain is low when the measurement error covariance is larger than the process error covariance. The new measurement $M_{k}$ and gain *G* are described as follows:

(7) $$M_{k}=Z-H\alpha_{k},$$

(8) $$G=\frac{{(P}_{k}H^{T})}{H.{(P}_{k}H^{T})+M_{e}},$$

where *Z*, *H*, and $M_{e}$ represent the transition, identity matrix, and measurement error covariance matrix, respectively. After estimating the Kalman gain *G*, the predicted state $\alpha_{k}$ and process error covariance matrix $P_{k}$ are updated in (9) and (10), respectively:

(9) $$\alpha_{k}=X\alpha_{k}+GM_{k},$$

(10) $$P_{k}=\left[\left(I-(GH\right)\right)+P_{k}].$$

Here, $M_{k}$ is the updated measurement which is obtained by subtracting the transition or measured matrix (*Z*) from the predicted state ($\alpha_{k}$) as described in (7). The update predicted state and process error covariance matrix in (9) and (10) will be used in the next time step.

### 3.3. Best Sensor Selection

The designed LSTM-DQN-epsilon-greedy system uses multiple sensors to track the target position. We consider one target at a particular time in a specific subarea as shown in Figure 2. The system operates in such a manner that it does not allow all sensors concurrently to track the target due to limited battery lifespan of the sensor devices. Therefore, the system intelligently adjudicates to select the best sensor using our proposed Deep RL method while the moving target arrives within that sensor’s range. The sensor with low energy consumption is considered the best sensor and is apportioned to acquire target position information. In the example shown in Figure 2, if the energy consumption of the four sensors (i.e., $S_{1}$, $S_{2}$, $S_{3}$, and $S_{4}$) are 6J, 5J, 7J, and 8J, respectively, then sensor $S_{2}$ is selected to track the target. In this way, we can conserve the energy of the other three sensors. As a result, the overall system capability has improved in a particular subarea.

### 3.4. Reinforcement Learning (RL)

The RL agent is used as a decision-maker to take the best action ($a_{t}$) from the set of possible actions over the current state ($s_{t}$). The RL agent does not learn with the labeled training dataset, but learns from its experience with environmental interaction. During environmental interaction at a particular time, the agent receives an immediate reward ($r_{t}$) and jumps to the next state ($s_{t+1}$). The entire process continues until the agent reaches the final state and begins a new episode after resetting the environment.

Tabular Q-learning (TQL) is a common model-free RL approach that is considered an off-policy algorithm because the Q-function learns from the interactive environment by taking random actions during exploration time [48]. Taking action with the help of exploration is essential because initially, the agent has no idea about the new state in an environment; therefore, the agent needs to explore the environment. After acquiring environmental experience by exploration, the agent can easily exploit the environment by utilizing the greedy strategy. The exploration and exploitation technique is also called the epsilon-greedy technique [19]. As a result that the TQL is a value-based method, the agent learning policy is utilized through the value function (Q-value) of state-action pairs. In TQL, the Q-value $Q\left(s_{t},\;a_{t}\right)$ of an individual action of a particular state is stored in a matrix called the Q-table, which is updated in each time step in (11),

(11) $$Q\left(s_{t},\;a_{t}\right)=\;Q\left(s_{t-1},\;a_{t-1}\right)+\;\partial \left(r_{t}+\;\gamma \max\left(Q\left(s_{t+1},a_{t+1}\right)\right)-Q\left(s_{t-1},a_{t-1}\right)\right),\;$$

where *∂* and γ∈[0,1] represent the learning rate and discount factor, respectively. Note that, $\partial \left(r_{t}+\;\gamma \max\left(Q\left(s_{t+1},a_{t+1}\right)\right)\right)$ denotes as discounted temporal difference (TD) target, which gives the maximum Q value of next state in (11). Further, to estimate the TD error during the training of Q-learning, we subtract the value of TD target from previous Q value $\left(Q\left(s_{t-1},a_{t-1}\right)\right)$. The learning rate is used, which tells how fast the Q-values are updated along with TD error. Moreover, the discount factor gives stability between immediate and upcoming or future rewards. If the discount factor is near to 1, then the reward will be more in the future. Otherwise, the system focuses on the immediate reward when the discount factor is near to 0. However, TQL has difficulty in extending the Q-table to a large environment, as it is only appropriate for a small environment. To extend the method to a large environment it is necessary for an agent to learn the value function with a Q-approximator instead of saving all values into a Q-table.

### 3.5. Deep-Q-Network

The DQN was introduced by Mnih et al. in [18] based on the Deep RL method with the help of a deep neural network, which is known as a Q-approximator. The Q-values of different actions are predicted by utilizing the Q-approximator in a particular state. In DQN, there is a possibility of a significant correlation between the data, forming the Q-approximator instability during the training period. Following this, experience replay memory and mini-batch techniques are utilized to obtain a stable Q-approximator. Experience replay memory (*E*) stores the experience $\;\left(s_{t},\;a_{t},\;{r_{t},\;s}_{t+1}\right)$ in each time step to re-utilize previous experiences multiple times. After storing each experience, the DQN uses the mini-batch technique to randomly sample data from the experience replay memory to converge the Q-approximator loss. It can also reduce the correlation between the samples and improve the agent’s learning performance. Moreover, we estimate the predicted and target Q-values with two different Q-approximators θ and $\theta^{\prime }$, respectively, to obtain a stable Q-approximator by optimizing the loss during the training period. The Q-approximator loss L(θ) is described as,

(12) $$L\left(\theta \right)={\left(r_{t}+\gamma \max\left(Q\left(s_{t+1},a_{t+1};\theta^{\prime }\right)\right)-Q\left(s_{t},a_{t};\theta \right)\right)}^{2}.$$

## 4. The Proposed LSTM-DQN-Epsilon-Greedy Method

### 4.1. Long Short-Term Memory-Based Q-approximator

**Figure 3:**
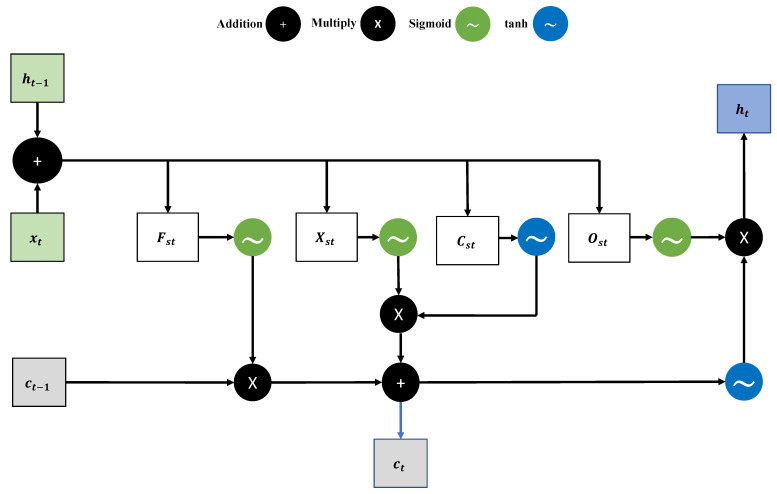
LSTM architecture.

In our proposed system, we use LSTM as a Q-approximator to select the best sensor. In our target tracking scenario, the position of the target is updated over time. The LSTM is a specific type of recurrent neural network (RNN) with the ability to learn long-term dependencies that can memorize and connect related patterns over a time-series input [22,23]. Moreover, another reason behind deploying LSTM for our designed system is that it works flawlessly in a dynamic environment because it depends on the gate operation. The gates regulate the information flow and can also decide which information should be stored or removed. The LSTM consists of four gates: forget ($F_{st}$), input ($X_{st}$), cell ($C_{st}$), and output ($O_{st}$) states. These four gates store the combined information of the previously hidden $\left(h_{t-1}\right)$ and the current input layer $\left(x_{t}\right)$ and apply the “sigmoid” operation to all gates except the cell state that is finally activated by “tanh” operation, as shown in Figure 3.

In the LSTM mechanism, when the forget state output is near 1, it keeps the data and transfers it to multiply with the previous cell state value ($C_{t-1}$). The input and cell state gates receive the same information as the forget state gate. After separately applying “sigmoid” and “tanh” operations to input and cell state gate, the outputs are multiplied with each other and added to the forget state output multiplying of the previous cell state value for acquiring a new cell state $\left(C_{t}\right)$. Finally, the output of the new cell state and output state gate after the sigmoid operation multiply with each other to obtain the new hidden state $\left(h_{t}\right)$.

### 4.2. Mini-Max Normalization-Based State Space

The proposed LSTM-DQN-epsilon-greedy model acts as an agent that takes the current state as the input. Estimated minimum distance leads to low energy consumption at a specific time. The sensor with the minimum distance and energy consumption is considered to be the best sensor for an individual area. Therefore, we organized our state with individual distances (i.e., $d_{S_{1}},d_{S_{2}},\ldots ,d_{S_{D}}$) between the target and sensors. The distance is measured at each time step by using the Euclidean distance formula in (13),

(13) $$d_{S_{D}}\left(t\right)=\;\sqrt{(P_{target_{xcord}}-P_{{xcord}_{S_{D}}}{)}^{2}\left(t\right)+\;(P_{target_{ycord}}-P_{{ycord}_{S_{D}}}{)}^{2}\left(t\right)},$$

where $P_{{xcord}_{S_{D}}}$, $P_{{ycord}_{S_{D}}}$, $P_{target_{xcord}}$, and $P_{target_{ycord}}$ are the positions of all the deployed sensors and the moving target in the two dimensional x-y plane. Furthermore, the position of any target is computed using the Kalman filter. Note that the state has different distance value ranges, which can create instability for the Q-approximator. Therefore, it is necessary to preprocess the state value by normalization before sending it to the LSTM Q-approximator [25]. We use the mini-max normalization method, which is represented as $state_{normalized}\left(t\right)=\frac{{(s}_{t}-\min\left(s_{t}\right))}{\max\left(s_{t}\right)-\min\left(s_{t}\right)}$ to scale the state between 0 and 1 to enhance the state quality before sending it to our proposed LSTM Q-approximator.

### 4.3. Epsilon-Greedy Discrete Action Space

The discrete action space (*A* = $\left\{A_{S_{1}},A_{S_{2}},\ldots ,A_{S_{D}}\right\}$) represents all the allocated sensors (i.e., $S_{1}$, $S_{2}$, …, $S_{D}$), respectively, in a defined area. The LSTM-DQN-epsilon-greedy agent selects the best sensor as an action that consumes minimum energy during target tracking. The energy consumption ($E_{con_{S_{D}}}$) of each sensor at time step (*t*) is estimated using (14), where $d_{S_{D}}$, $pow_{Sensor}$, and $t_{track}$ indicate the distance value between a particular sensor ($S_{D}$) and the target, the working mode sensor power, and time to track the target in a single area, respectively. Similarly, we measured the energy consumption for the other *N* areas. Note that the energy consumption of all sensors is stored in an array as ($E_{con_{all}}$) in (15). Furthermore, the selected sensor energy consumption ($E_{con_{action}}$) and minimum energy consumption ($E_{con_{min}}$) are obtained from (16) and (17). Finally, we estimate the total energy consumption ($E_{con_{total}}$) and energy savings in a particular observation using (18) and (19), respectively:

(14) $$E_{con_{S_{D}}}\left(t\right)=\;d_{S_{D}}\left(t\right)\times pow_{sensor}\left(t\right)\times t_{track}\left(t\right),$$

(15) $$E_{con_{all}}\left(t\right)=\;E_{con_{S_{D:1∼D}}}\left(t\right),$$

(16) $$E_{con_{action}}\left(t\right)=\;E_{con_{all}}\left[A_{S_{D}}\right]\left(t\right),$$

(17) $$E_{con_{min}}\left(t\right)=\;\min\left(E_{con_{all}}\left(t\right)\right),$$

(18) $$E_{con_{total}}\left(t\right)=\;\sum_{S_{D=1}}^{D}E_{con_{S_{D}}}\left(t\right),$$

(19) $$E_{save}\left(t\right)=\;E_{con_{total}}\left(t\right)-E_{con_{action}}\left(t\right).$$

**Figure 4:**
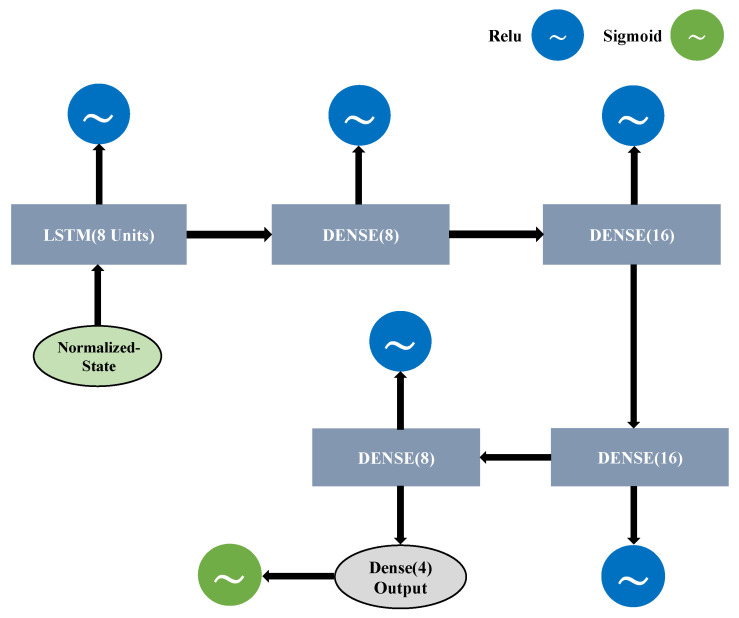
Proposed LSTM Q-approximator.

We use epsilon-greedy as an action-selection strategy in the designed system because it is suitable for the discrete action space. In the epsilon-greedy approach, initially, the agent takes a random action to explore the environment through the epsilon method. There are three key parameters: maximum-epsilon ($\varepsilon_{max}$), minimum-epsilon ($\varepsilon_{min}$), and epsilon-decay ($\varepsilon_{decay}$) that are considered to fix the epsilon period. First, it begins with the maximum-epsilon value and then decays with an absolute epsilon-decay value at each time step. The epsilon period is completed when the value of epsilon reaches the minimum-epsilon. Subsequently, the agent greedily exploits the environment to take suboptimal action with the proposed LSTM Q-approximator, as shown in Figure 4.

The rectified linear unit (ReLU) is used in the first three layers, whereas the sigmoid activation function works at the output layer. The ReLU is used to obtain the unbounded positive outcome, whereas sigmoid is used in the output layer to obtain a positive bounded outcome between 0 and 1. Moreover, the LSTM Q-approximator predicts the Q-values for all possible actions, which are defined in the action space. Finally, the agent selects the suboptimal action with the highest action-Q value that is obtained by $\arg\max(Q(state_{normalized_{t},a_{t};\theta }))$.

### 4.4. Binary-Based Reward Space

**Figure 5:**
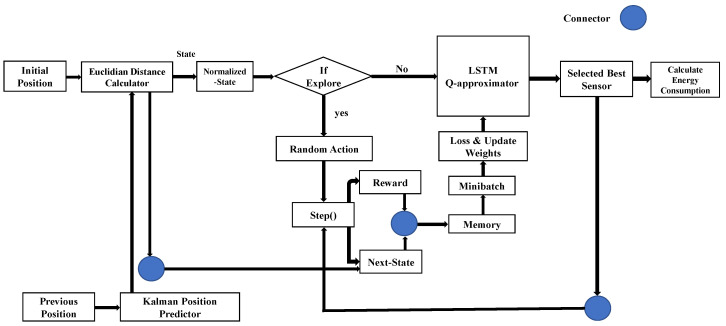
Proposed LSTM-DQN-epsilon-greedy system architecture.

The primary goal of our proposed system is to maximize the cumulative rewards after a certain number of steps; therefore, it needs to generate a suitable reward mechanism to improve the agent action. The binary reward function is used in the proposed system design as follows:

$$r_{t}=\left\{\begin{array}{cc} 1 & \mathrm{if}\;E_{con_{action}}\;=\;E_{con_{min}}\\ 0 & \mathrm{if}\;E_{con_{action}}\;\neq \;E_{con_{min}}, \end{array}\right.$$

where $r_{t}$ is the reward at time *t*; further, if the energy $E_{con_{action}}$ is equal to $E_{con_{min}}$, it returns 1; otherwise, the output will be 0. The proposed LSTM-DQN-epsilon-greedy system architecture and algorithm are shown in Figure 5 and Algorithm 1, respectively.
**Algorithm 1:** The proposed LSTM-DQN-epsilon-greedy algorithm.
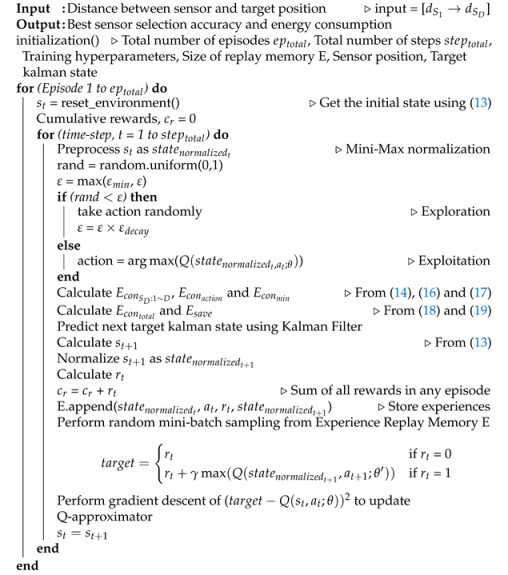



## 5. Simulation and Results

### 5.1. Environment Setup, Hyper Parameters, and Evaluation Metrics

To evaluate our proposed system, a simulation platform with moving target observation of 16 sensor devices is considered with four subareas, where each subarea consists of 200 m × 200 m. We allocated four sensors in each subarea, and each sensor can cover an area of up to 50 m × 50 m. Thus, 16 sensors cover a total area of 800 m × 800 m. Furthermore, the distance between each sensor was the same in each subarea. We assume one target in a particular subarea and extend it to four targets in four different subareas at a specific time. The environmental details are listed in Table 3.

**Figure 6:**
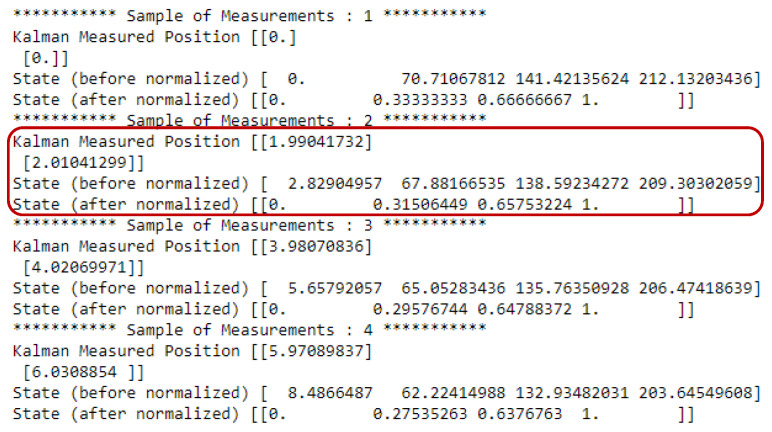
Some samples of measurement during simulation.

**Figure 7:**
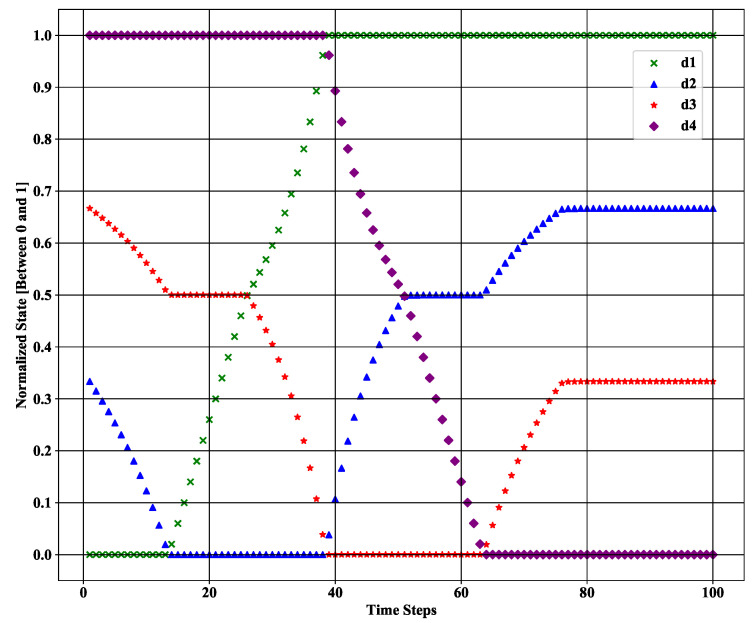
Normalized state value for each time step during the experiment.

During our simulation, we assumed that the total number of episodes was 500, where each episode consisted of 100 time steps. In each time step, the target positions are updated using the Kalman filter method. Thus, we can utilize 100 different states for our proposed LSTM-DQN-epsilon-greedy system in one episode. Figure 6 shows a sample of data during the experiment that contains measured values. Moreover, Figure 7 shows a sample of different state values in one area after applying the normalization (i.e., mini-max normalization, which was described in Section 4.2) at the time of the experiment. Here, d1, d2, d3, and d4 represent the normalized distance values between the four sensors and the target. The normalized state was near zero when the moving target passed near a particular sensor. Conversely, the particular distance values were greater than 0 and gradually increased to 1 when the target moved far behind the sensor. The figure clearly shows that the initial value of d1 (i.e., the distance between the first sensor and the target) is zero as the target moves very close to the first sensor. The same is true for the other sensor distance values during the simulation period.

Note that we restart each episode when the number of steps reaches 100, and targets again start moving from the initial position. Moreover, some useful hyperparameters were set during the training session, as presented in Table 4. These parameters are used to tune the proposed LSTM-DQN-epsilon-greedy scheme to achieve a more stable output. These hyperparameter values were chosen by a trial and error process. We performed simulations using Python 3.7.7 [49]. TensorFlow 2.0.0 and Keras 2.3.1 were used to implement the LSTM Q-approximator [50,51].

The mathematical formulas to evaluate our proposed method are shown in Table 5.

### 5.2. Results

#### 5.2.1. Cumulative Rewards

**Figure 8:**
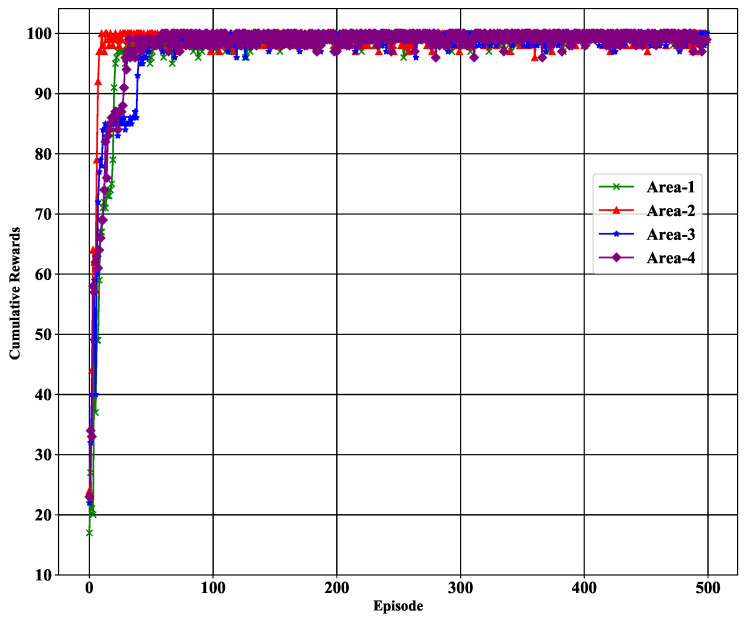
Cumulative rewards for each area.

In our proposed LSTM-DQN-epsilon-greedy method, we first measure the cumulative rewards ($c_{r}$) as shown in Table 5 for each episode. The estimation of the cumulative reward is important because it indicates the agent’s learning performance during interaction with the target tracking environment. The proposed agent receives a reward of 1 when the agent successfully selects the best sensor, as discussed briefly in Section 4.3 and Section 4.4. In Figure 8, the cumulative reward is shown per episode for each subarea. It shows that the cumulative reward is less than 35 for each subarea and does not reach the highest value in the first two episodes (200 steps), as it initially explores the environment. In general, the exploration duration depends on the epsilon parameter values (i.e., $\varepsilon_{max}$, $\varepsilon_{min}$, and $\varepsilon_{decay}$) given in Table 3. 

Following the exploration stage, the proposed agent starts exploiting the environment through a greedy approach for selecting the best sensor to track the target. In this case, the agent selects the suboptimal action based on the maximum predicted action-Q value. During the greedy process, the cumulative reward gradually increased after the second episode for all subareas. As we have 100 different states in each episode, therefore, the maximum cumulative reward is 100. The proposed agent needs to obtain the highest cumulative reward as early as possible to reduce the energy consumption of the sensor. With the proposed method, the highest cumulative reward up to 100 was achieved before reaching 100 episodes for all subareas. The flow of maximum cumulative rewards is significantly stable, showing outstanding performance while selecting the best sensor.

#### 5.2.2. Best Sensor Selection Accuracy

**Figure 9:**
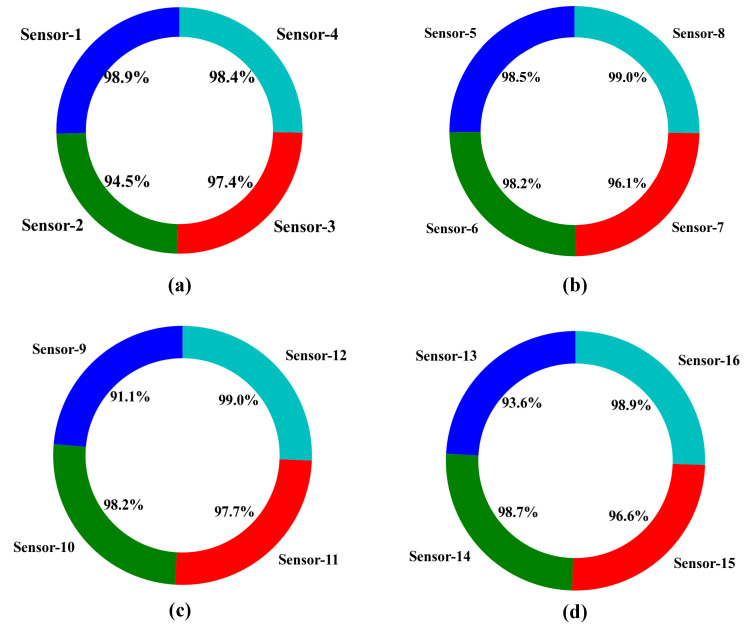
Best sensor selection accuracy: (**a**) Sensor selection accuracy for Area 1; (**b**) Sensor selection accuracy for Area 2; (**c**) Sensor selection accuracy for Area 3; (**d**) Sensor selection accuracy for Area 4.

As a result that sensors have a limited battery lifetime, it is essential to reduce energy consumption as much as possible. In the proposed scheme, the system selects the four best sensors at a particular time within an area of 800 m × 800 m divided into Areas 1, 2, 3, and 4, as shown in Figure 2. Due to having different ranges of state values, it is difficult to achieve better accuracy of best sensor selection by our proposed LSTM Q-approximator. As a result, our proposed agent selects the energy-efficient sensor based on normalized state, which has been described in Section 4.2. Furthermore, the accuracy of selecting the best sensor affects energy consumption during the tracking target because the best sensor selection is based on the minimum energy consumption described in Section 4.3. Figure 9 shows the best sensor selection accuracy for the 16 sensors (as formulated in Table 5). This demonstrates that the proposed LSTM-DQN-epsilon-greedy system has a significant accuracy of approximately 99% for sensors 1, 8, 12, 14, and 16. Similarly, the system achieved an accuracy of 98% for sensors 4, 5, 6, and 10. Moreover, the proposed system provides more than 90% accuracy in the case of all other sensors, leading to promising results.

### 5.3. Comparative Analysis

The proposed LSTM-DQN-epsilon-greedy system is also compared with three benchmark schemes: LSTM-DQN-softmax, Dense-DQN-epsilon-greedy, and Dense-DQN-softmax in terms of average cumulative reward, loss convergence, average best sensor selection accuracy, and cumulative energy consumption. In DQN, the LSTM and dense-based Q-approximator are used frequently for the dynamic environment. However, LSTM exhibits better performance in handling such an environment because of memory features. We also utilized different action-selection strategies (e.g., epsilon-greedy and softmax) compared with our scheme.

#### 5.3.1. Average Cumulative Reward

**Figure 10:**
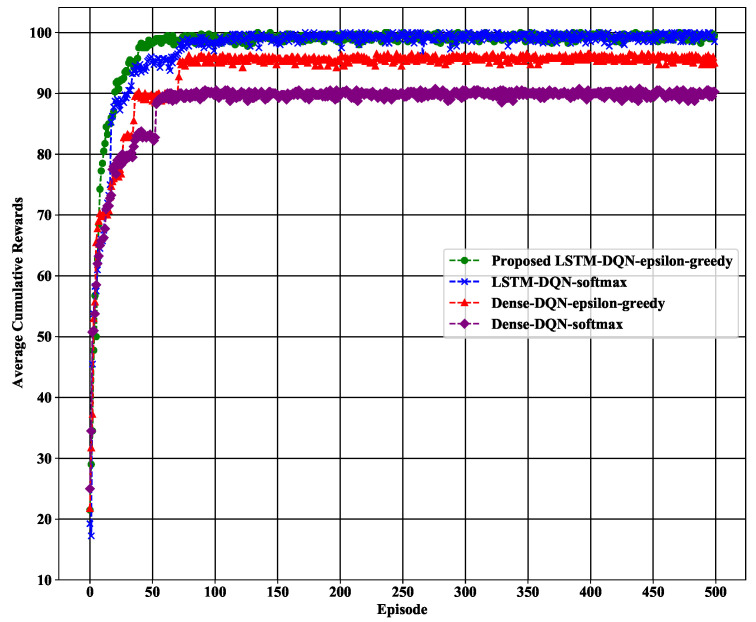
Average cumulative rewards per episode.

The key designed method deployment objective is to increase the average cumulative reward ($avg_{c_{r}}$) as described in Table 5 to measure the agent’s performance. Figure 10 shows the average cumulative reward per episode for the four DQN-based schemes. The figure shows that our proposed model and the LSTM-DQN-softmax model both achieved the highest average cumulative reward, which was up to 100 during the simulation period. However, LSTM-DQN-epsilon-greedy reached achieved the highest value faster in 63 episodes compared to the LSTM-DQN-softmax, which reached that level in 115 episodes. The efficiency of our proposed system is that the epsilon-greedy action selection strategy directly learns from the action-Q-value function, which is suitable for discrete action space.

Furthermore, the comparison has been extended to the other two Dense-DQN-based schemes: Dense-DQN-epsilon-greedy and Dense-DQN-softmax. The performance of both LSTM-DQN-based approaches is better than that of Dense-DQN methods because of the long-term memory dependencies. Therefore, both the Dense-DQN-epsilon-greedy and Dense-DQN-softmax schemes are unable to reach the highest average cumulative reward over the entire 500 episodes, and the average cumulative reward increase of both methods is much slower than the proposed LSTM-DQN-epsilon-greedy scheme.

#### 5.3.2. Loss Convergence

**Figure 11:**
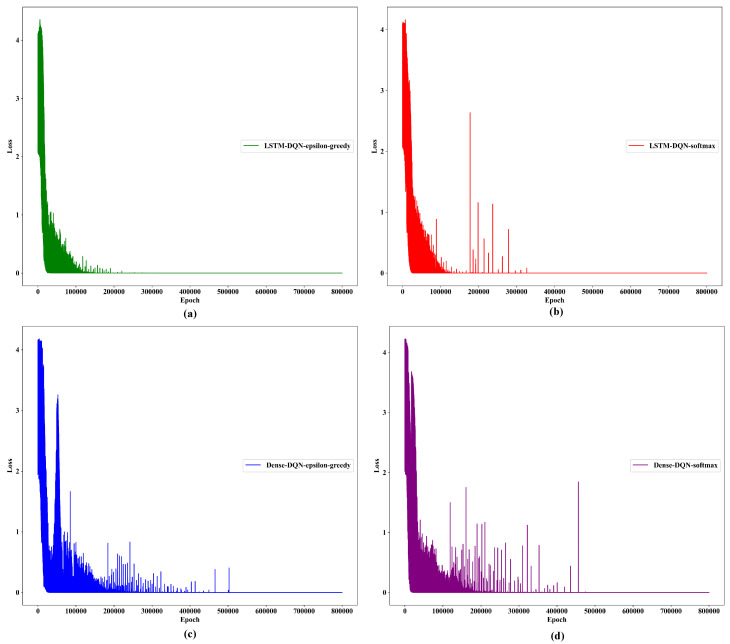
Loss convergence per epoch during training: (**a**) loss convergence for proposed epsilon-greedy-LSTM-DQN; (**b**) loss convergence for softmax-LSTM-DQN; (**c**) loss convergence for epsilon-greedy-Dense-DQN; (**d**) loss convergence for softmax-Dense-DQN.

The loss convergence depreciation to the minimum level is also vital, along with the system stability. To estimate the loss of our proposed Q-approximator, we use categorical crossentropy because it is suitable for multiclass classification problems (as presented in Table 5). The proposed LSTM-DQN-epsilon-greedy system signifies good convergence behavior around 200,000 epochs and is more stable, as illustrated in Figure 11. Moreover, the LSTM-DQN-softmax convergence also appeared around 200,000 epochs, but was less stable than our proposed scheme. Furthermore, Dense-DQN-epsilon-greedy and Dense-DQN-softmax methods show unstable behavior and converge at 500,000 epochs, which is time-consuming. Therefore, the proposed LSTM-DQN-epsilon-greedy algorithm is efficient and stable, leading to promising results.

#### 5.3.3. Average Best Sensor Selection Accuracy

**Figure 12:**
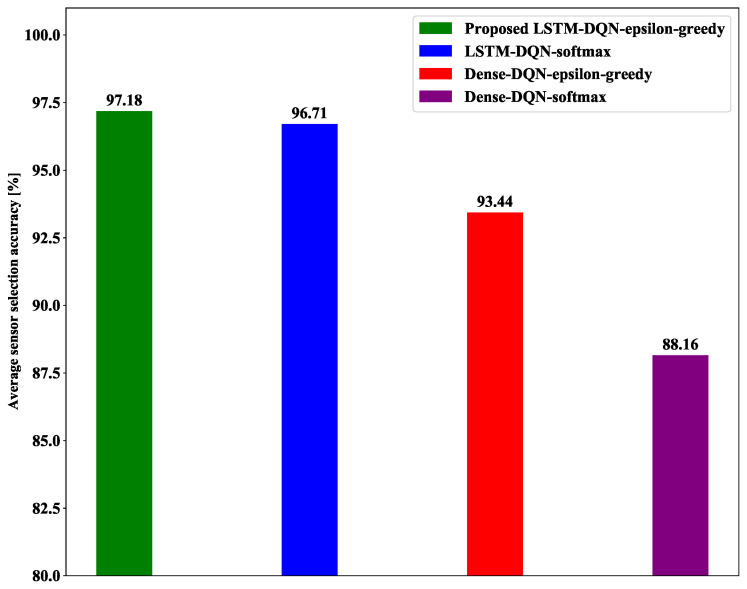
Average best sensor selection accuracy.

In this section, we compared the average best sensor selection accuracy (as described in Table 5) of the proposed system with that of the other three DQN methods, as presented in Figure 12. In our study, the agent selects the best sensor that has minimum energy consumption when the target moves in any particular area. The critical task is to significantly enhance the best sensor selection accuracy to reduce the average energy consumption. As shown in Figure 12, the proposed system agent selects the best sensor with a slightly higher average accuracy than LSTM-DQN-softmax. Furthermore, the proposed LSTM-DQN-epsilon-greedy scheme achieved significantly higher best sensor selection accuracy than the Dense-DQN-epsilon-greedy and Dense-DQN-softmax methods.

#### 5.3.4. Average Cumulative Energy Consumption

Our designed system was also utilized to reduce the average cumulative energy consumption while tracking the target. We already mentioned in Section 5.3.1 and Section 5.3.3, that a higher average cumulative reward effectively enhances the best sensor selection accuracy and reduces the average cumulative energy consumption. The average cumulative energy consumption ($avg_{E_{con}}$) is obtained using a formula, which is shown in Table 5. 

**Figure 13:**
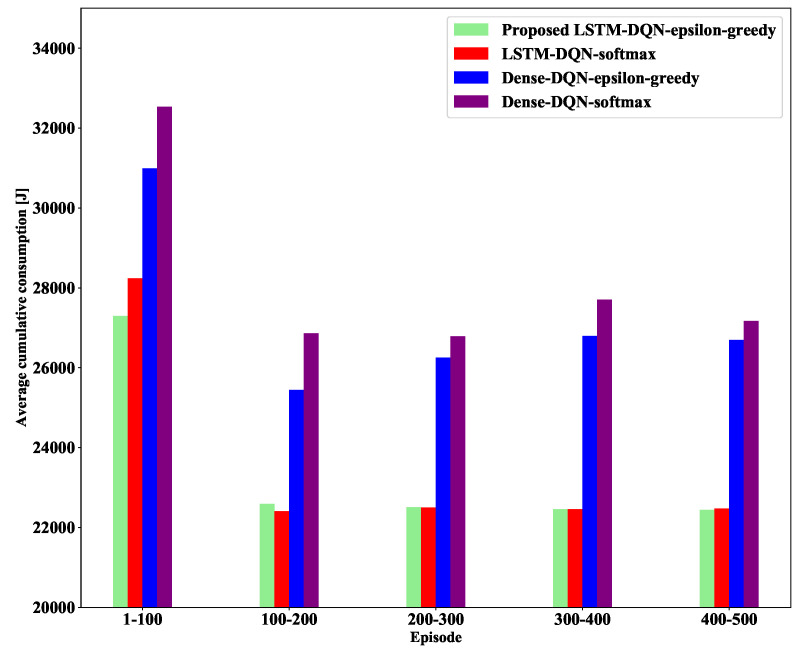
Average cumulative energy consumption.

Figure 13 shows the average cumulative energy consumption in 500 episodes. It can be observed from the figure that the average cumulative energy consumption for each method is higher, particularly in the first 100 episodes. The reason behind it is that initially, the agent has no experience with the environment. However, as the number of episodes increases, the average cumulative energy consumption decreases significantly for both LSTM-DQN- and Dense-DQN-based schemes. 

In contrast, both LSTM-DQN-epsilon-greedy and LSTM-DQN-softmax methods have much lower average cumulative energy consumption compared to Dense-DQN-epsilon-greedy and Dense-DQN-softmax because the LSTM Q-approximator can regulate the information flow in memory in the long and short term. Furthermore, both the LSTM-DQN-epsilon-greedy and LSTM-DQN-softmax schemes approximately reduce the same average cumulative energy consumption in each episode except 1 to 200. However, the proposed LSTM-DQN-epsilon-greedy method shows a faster and better reduction of the average cumulative energy consumption than LSTM-DQN-softmax, particularly in the first 100 episodes. Thus, our designed LSTM-DQN-epsilon-greedy method significantly reduced the average cumulative energy consumption compared to the other three methods by selecting the best energy-efficient sensor in our designed target tracking environment.

## 6. Conclusions and Future Directions

Sensors are widely used in IoT applications (e.g., tracking and attaining target location information). In such scenarios, energy consumption optimization is a critical challenge because of the sensor battery lifespan. For this reason, an adequate learning method with Deep RL has been proposed to overcome the problem of energy consumption. The proposed idea is based on selecting the best sensor with minimum energy using the proposed Deep RL agent at a particular time to collect the target location information. The Kalman filter and LSTM-DQN-epsilon-greedy algorithms have been utilized to predict the target position and best sensor selection, respectively. Furthermore, we compared our proposed LSTM-DQN-epsilon-greedy system with the other three benchmark schemes: LSTM-DQN-softmax, Dense-DQN-epsilon-greedy, and Dense-DQN-softmax. A comparative analysis was performed in terms of average cumulative reward, loss convergence, average best sensor selection accuracy, and cumulative energy consumption. Our proposed LSTM-DQN-epsilon-greedy method addresses the problem of best sensor selection and converges the energy consumption issue efficiently, which is significantly improved in our tracking environment than the other three methods. 

The limitation of the proposed scheme is that we only considered the linear target information using the Kalman filter. However, the target position can be non-linear, which is out of scope of this study. Moreover, the framework is unable to track multiple targets in one subarea at a particular time. To track the multiple targets information simultaneously, we need to activate more than one sensor in one subarea. The framework will be extended to use multi-agent-based Deep RL in future work to control the multiple sensors efficiently. Finally, the system could also leverage hardware in the future to carry out real-time hardware experimentation. 

## Figures and Tables

**Table 1:** Related work that use RL-based methods to reduce the energy consumption of the sensor.

Study	RL-Based Methods	Action-Selection	Solution	Evaluation Metrics
[39]	SARSA (λ)	epsilon-greedy	sensor scheduling	energy consumption
[40]	Q-table with stacked autoencoder	epsilon-greedy	transmission scheduling	average power consumption and system utility
[27]	DQN, DDPG with LSTM	epsilon-greedy and softmax	radius adjustment of the activated area	average cumulative rewards and energy consumption
Proposed method	LSTM-DQN	epsilon-greedy and softmax	best sensor selection	average cumulative rewards, loss convergence, average best sensor selection accuracy, and average average cumulative energy consumption

**Table 2:** Kalman filter parameters.

Symbols	Description
$\alpha_{0}$	Initial state matrix
$P_{0}$	Initial process covariance matrix
$\alpha_{k-1}$	Previous state matrix
$M_{k}$	Measurement input
*G*	Kalman gain
$Acc_{k}$	Control variable matrix
$P_{k-1}$	Previous process covariance matrix
$N_{k\alpha }$	Predicted noise matrix
$N_{kp}$	Process noise matrix
X,Y,Z	Transition matrix
$M_{e}$	Measurement error covariance matrix
*H*, *I*	Identity matrix

**Table 3:** Details of the proposed environment.

Parameters	Value
Total number of subareas (*N*)	4
Size of a subarea ($X_{N}$)	200 m × 200 m
Number of sensors in a subarea ($X_{N}$)	4
Total number of sensors in 4 subareas	16
Each sensor tracking range	50 m × 50 m
Power of sensor in working mode ($pow_{sensor}$)	5 watts
Tracking time of sensor per meter ($t_{track}$)	2 s
Number of target (each subarea)	1
Total number of targets in 4 subareas	4
Targets initial positions	[0, 0]–[200, 200]–[400, 400]–[600, 600]
Target initial velocity	[0.1 m/s, 0.2 m/s]
Target initial acceleration	[5 m/s^2^, 5 m/s^2^]

**Table 4:** Hyperparameters for LSTM-DQN-epsilon-greedy during training.

Hyperparameter	Value
Optimizer	adam
Loss	categorical crossentropy
Batch Size	16
Size of experience replay memory (*E*)	50
Learning rate (*∂*)	0.001
Discount factor (γ)	0.9
Maximum epsilon ($\varepsilon_{max}$)	1
Minimum epsilon ($\varepsilon_{min}$)	0.01
Epsilon decay ($\varepsilon_{decay}$)	0.995

**Table 5:** A list of evaluation metrics.

Definition	Formula
Cumulative rewards (described in Section 5.2.1)	$c_{r}$ = $\sum_{t=1}^{101}r_{t}$
Best sensor selection accuracy. here, $T_{Best_{A_{S_{D}}}}=$ total number of predicted best sensor and $T_{Wrong_{A_{S_{D}}}}=$ total number of predicted wrong sensor (described in Section 5.2.2)	$Acc_{S_{D}}=(\frac{T_{Best_{A_{S_{D}}}}}{T_{Best_{A_{S_{D}}}}+T_{Wrong_{A_{S_{D}}}}})\times 100$
Average cumulative reward. here, ep denotes the episode and $X_{1}$, $X_{2}$, $X_{3}$, and $X_{4}$ are four system subareas. (described in Section 5.3.1)	$avg_{c_{r}}=\sum_{ep=1}^{501}\frac{c_{r_{X_{1}}}(ep)+c_{r_{X_{2}}}(ep)+c_{r_{X_{3}}}(ep)+c_{r_{X_{4}}}(ep)}{4}$
The categorical crossentropy loss convergence. here, $y_{j}=r_{t}+\gamma \max\left(Q\left(s_{t+1},a_{t+1};\theta^{\prime }\right)\right)$, $y_{j}^{\prime }=Q\left(s_{t},a_{t};\theta \right)$ and *s* = size of the action space (described in Section 5.3.2)	$CC_{Loss}=-\sum_{j_{s}=1}^{s}y_{j}\log(y_{j}^{\prime })$
Average best sensor selection accuracy here, D is the total number of sensor (described in Section 5.3.3)	$avg_{Acc}=\frac{\sum_{SD=1}^{D}Acc_{S_{D}}}{D}$
Average cumulative energy consumption (described in Section 5.3.4)	$avg_{E_{con}}=\sum_{ep=1}^{501}\frac{E_{{con}_{{action}_{X_{1}}}}(ep)+E_{{con}_{{action}_{X_{2}}}}(ep)+E_{{con}_{{action}_{X_{3}}}}(ep)+E_{{con}_{{action}_{X_{4}}}}(ep)}{4}$

## Data Availability

Not applicable.
